# Characterization and fungicide sensitivity of *Colletotrichum godetiae* causing sweet cherry fruit anthracnose in Guizhou, China

**DOI:** 10.3389/fmicb.2022.923181

**Published:** 2022-09-27

**Authors:** Keqin Peng, Yintao Pan, Tingjun Tan, Xiangyu Zeng, Meiling Lin, Shuang Jiang, Zhibo Zhao, Fenghua Tian, Xiaosheng Zhao

**Affiliations:** Department of Plant Pathology, College of Agriculture, Guizhou University, Guiyang, China

**Keywords:** *Cerasus pseudocerasus*, plant disease, *Colletotrichum*, multi-gene, sweet cherry fruit anthracnose

## Abstract

Sweet cherry is an important fruit crop with high economic and ornamental value in China. However, cherry fruit anthracnose, caused by *Colletotrichum* species, greatly impacts cherry yield and quality. Here, we surveyed cherry anthracnose in Guizhou, China from 2019–2020. Necrotic sweet cherry fruits were collected from different areas in Guizhou and examined. A total of 116 *Colletotrichum* strains were isolated from these symptomatic fruits. Based on the morphological characteristics of the isolates and phylogenetic analyses of concatenate internal transcribed spacer (ITS) region and *ACT*, *CHS-1*, *GAPDH*, *TUB2,* and *HIS3* genes, the pathogen responsible for causing sweet cherry anthracnose was identified as *Colletotrichum godetiae*. Pathogenicity tests were conducted by inoculating healthy sweet cherry fruits with spore suspensions of the fungal pathogen, and Koch’s postulates were confirmed by pathogen re-isolation and identification. The Q-1 isolate showed different sensitivities to 13 fungicides, exhibiting seven different modes of action, and its EC_50_ values ranged from 0.04 to 91.26 μg ml^−1^. According to that, the sensitivity of 20 isolates from different samples to ten fungicides with better performance, were measured. The results showed that 6 of the 10 fungicides (difenoconazole, propiconazole, prochloraz-manganese, pyraclostrobin, trifloxystrobin-tebuconazole, and difenoconazole-azoxystrobin) all showed higher sensitive to the 20*\u00B0C. godetiae* isolates, and no resistance groups appeared. Its EC50 values ranged from 0.013 to 1.563 μg ml^−1^. In summary, this is the first report demonstrating that *C. godetiae* causes sweet cherry anthracnose and the results of this study provide insights into how sweet cherry anthracnose could be effectively controlled in China.

## Introduction

Chinese sweet cherry (*Cerasus pseudocerasus* Lindl.; Rosaceae) is an important native fruit crop with high economic and ornamental value (Chen et al., 2012, 2013b). ‘Manaohong’, approved by Guizhou Provincial Variety Approval Committee in 2011, is an unique local sour cherry variety in Guizhou Province, China. Cherry fruits are rich in vitamins, niacin, phenolic compounds, and minerals (Kim et al., 2005; Zhang et al., 2008). Sweet cherry cultivation is one of the 12 most important agricultural sectors that contribute to the development of characteristic agriculture in Guizhou. With the expansion of ‘Manaohong’ cherry cultivation and the increase in temperature and rainfall, the occurrence of fungal disease of cherry fruits has significantly increased in Guizhou.

Anthracnose is a widespread disease that reduces crop yield and quality, resulting in great economic losses. *Colletotrichum*, the causal agent of anthracnose, is one of the top 10 fungal genera of economic and scientific importance (Dean et al., 2012). *Colletotrichum* is the only genus in the Glomerellaceae family (order Glomerellales, class Sordariomycetes) (Wijayawardene et al., 2018, 2020). Species of *Colletotrichum* are known as pathogens (causing anthracnose and postharvest fruit rot in plants), endophytes (producing a range of secondary metabolites), and saprobes (Bhunjun et al., 2021). Anthracnose, caused by *Colletotrichum* spp., is an important disease that seriously threatens the production of sweet cherry. Although *Colletotrichum* spp. are known to infect leaves and young shoots of cherry trees, they most frequently infect cherry fruits at various developmental stages (Børve and Stensvand, 2006a, 2013; Børve et al., 2010). For example, on the sweet cherry fruits infected by *Colletotrichum acutatum* in Norway, the initial symptoms of anthracnose on young fruit include dark brown spots, which later spread to the whole fruit and block fruit development. On mature fruits, the disease lesions are sunken and dark brown, and sticky piles of orange/yellow spores are formed on the lesion. Damage to the fruit reduces the yield and quality of sweet cherry, causing great economic losses (Børve and Stensvand, 2013).

A clear understanding of *Colletotrichum* species involved in sweet cherry anthracnose is essential for disease management. The genus *Colletotrichum* is divided into 14 species complexes, which comprise approximately 189 species (Jayawardena et al., 2021). In previous studies, morphological characterization and molecular characterization were used to accurately identify *Colletotrichum* species (Cai et al., 2011). Most of the *Colletotrichum* spp. displayed an unique colony color, mycelial growth rate, and size and shape of conidia and appressoria, which were used as key morphological traits for preliminary identification (Damm et al., 2012). The *Colletotrichum* spp. were further distinguished based on molecular data, including two intergenic regions, internal transcribed spacer (ITS) region and the intergenic region between *apn2* and *MAT1-2-1* genes (ApMAT), and partial DNA sequences of five genes, namely actin (*ACT*), chitin synthase (*CHS-1*), histone 3 (*HIS3*), glyceraldehyde-3-phosphate dehydrogenase (*GAPDH*) and β-tubulin (*TUB2*) (Damm et al., 2012; Sharma et al., 2013). Based on this method, a growing number of *Colletotrichum* spp. causing cherry anthracnose has been reported. For example, four C*olletotrichum* species, *C. aenigma* based on ITS, *DAPDH*, *ACT*, *TUB2, and CHS-1* genes (Beijing city; Chethana et al., 2019), *C. pseudotheobromicola* sp. nov. based on ITS, *DAPDH*, *ACT*, *TUB2* and *CHS-1* genes (Beijing city; Chethana et al., 2019), *C. liaoningense* based on ITS, *DAPDH*, *ACT*, *TUB2* and *CHS-1* genes (Shandong province; Liu et al., 2021) and *C. fructicola* based on ITS, *DAPDH*, *ACT*, *TUB2,* and *CHS-1* genes (Zhejiang province; Tang et al., 2021), causing leaf spot on cherry, have been reported in China. In Brazil, *Colletotrichum theobromicola* was determined to cause necrotic and sunken spots on Barbados cherry fruit (Bragança et al., 2014). In southwestern Norway, *Colletotrichum acutatum* was reported to infect sweet cherry leaves (Børve et al., 2010), and overwinter on the buds (Børve and Stensvand, 2006a; Stensvand et al., 2017) and shoots (Stensvand et al., 2017) of sweet cherry, thus serving as the primary source of inoculum for more infections in the growing season. Among the above six pathogens, *C. aenigma*, *C. pseudotheobromicola*, *C. fructicola,* and *C. theobromicola*, were classified into the *C. gloeosporioides* species complex; *C. acutatum* was assigned to the *C. acutatum* species complex; and *C. liaoningense* was determined as a singleton species (Jayawardena et al., 2016).

Although the application of fungicides has adverse effects on the environment, chemical control is still the most effective measure for controlling the anthracnose disease. Recently, a number of studies reported the emergence of fungicide resistant strains of *Colletotrichum* species. In Hainan province, *C. gloeosporioides* strains highly resistant to carbendazim were isolated from mango, litchi, and longan (Zhang et al., 2014). Moreover, *C. gloeosporioides*, which causes grape ripe rot, showed a resistance to thiophanate-methyl and diethofencarb (Chen et al., 2013a). In other studies, fungicides were shown to be highly effective in controlling the anthracnose disease. For example, dithianon was effective in controlling anthracnose in sweet and sour cherry in Norway (Børve and Stensvand, 2006b). Methyl benzimidazole carbamate (MBC) and demethylation inhibitor (DMI) fungicides were used against *Colletotrichum* recently (Chen et al., 2016). In the USA, quinone outside inhibitors (QoIs) are the most common fungicides used in commercial strawberry fields for controlling anthracnose caused by *Colletotrichum* spp. (Forcelini et al., 2017). Understanding the sensitivity of *Colletotrichum* spp. to various fungicides may have significant implications on the effective management of this disease. However, the fungicide sensitivities of *Colletotrichum* isolates causing sweet cherry anthracnose in China remain unknown.

In the present study, we isolated *Colletotrichum* species associated with sweet cherry anthracnose in Guizhou province, and identified these species based on their morphological traits and multilocus phylogeny. Then, the pathogenicity of *Colletotrichum* species was determined by the artificial inoculation of sweet cherry fruits. Finally, we determined the sensitivities of the *Colletotrichum* isolates to different fungicides. The results of these analyses provide important information that could be used to develop an effective strategy for controlling sweet cherry fruit anthracnose.

## Materials and methods

### *Colletotrichum* strain isolation

During 2019–2020, anthracnose disease investigations were conducted on sweet cherry orchards in four regions of China’s Guizhou Province, namely Bijie (26°19′N, 106°46′E), Guiyang (26°19′N, 106°46′E), Liupanshui (26°18′N, 104°51′E) and Qianxinanzhou (25°56′N, 107°18′E). Through the statistics of diseased plant rate, we found that its incidence had reached 10 to 20%, hindering cherry fruit industry development. Therefore, to clarify the cause of the disease, we collected about 40 diseased sweet cherry fruits at different developmental stages from diseased orchards in these four regions.

To isolate the disease-causing pathogen, the infected fruits were washed with tap water, dried on absorbent paper, and then surface-sterilized by rubbing the surface of the lesion three times with a 75% ethanol-soaked cotton ball. The diseased tissue was crushed, immersed in sterilized water, and then subjected to gradient dilution, Low-titer spore suspensions were spread on a potato dextrose agar (PDA) plate (Yuan et al., 2021). It was then placed in the dark at 25°C for 4 days, and the single colonies that grew were picked into new PDA plates. The pure colonies were soaked in 20% (v/v) glycerol at −70°C for long-term storage (Kim et al., 2020). The isolated strains were classified according to the sampling location and strains with the same morphology, and 20 isolates were chosen for further study.

### Morphological characterization

The colony color of each isolate was recorded after culturing on PDA at 25°C for 6 days. Mycelial plugs (5 mm) excised from the margin of 6-day-old colonies were placed at the center of each PDA plate (total five plates), and cultured in darkness at 25°C. The diameter of each colony was measured by cross direction, and its daily growth rate was calculated. The experiment was repeated three times. A small amount of mycelia was scraped from 10-day-old colonies to observe the mycelial appressorium. Hyphal tips were sampled from the agar, transferred to fresh PDA plates, and incubated at 25°C for 10 days to induce the formation of conidia. Appressoria were induced as described previously (Cai et al., 2009). Using a compound light microscope (Zeiss Scope 5 with camera AxioCam 208 color), the shape and size of mycelial appressorium, conidia, and appressorium were recorded and measured.

### DNA extraction and sequencing

Genomic DNA was extracted from the mycelia of 6-day-old colonies cultured on PDA plates using the CWBIOTECH Plant Genomic DNA Kit (Changping, Beijing, China). The ITS region of the rDNA gene cluster, a 200-bp intron of the *GAPDH* gene, and partial sequences of *CHS-1*, *ACT*, *TUB2,* and *HIS3* genes were amplified from the genomic DNA using ITS4/ITS5, GDF1/GDR1, CHS-79F/CHS-354R, ACT-512F/ACT-783R, T1/T2, and CYLH3F/CYLH3R primer pairs, respectively (Crous et al., 2004; Damm et al., 2012; López-Moral et al., 2017). The sequences of primers used in this study are listed in Table 1.

**Table 1:** List of primers used in this study.

Gene or DNA region	Primer	DNA sequence (5′-3′)	Reference
ITS	ITS4	TCCTCCGCTTATTGATATGC	White et al. (1990)
	ITS5	GGAAGTAAAAGTCGTAACAAGG	
*GAPDH*	GDF	GCCGTCAACGACCCCTTCATTGA	Templeton et al. (1992)
	GDR	GGGTGGAGTCGTACTTGAGCATG	
*CHS-1*	CHS-79F	TGGGGCAAGGATGCTTGGAAGAA	Carbone and Kohn (1999)
	CHS-354R	TGGAAGAACCATCTGTGAGAGTTG	
*TUB2*	T1	AACATGCGTGAGATTGTAAGT	O’Donnell and Cigelnik (1997)
	T2	TAGTGACCCTTGGCCCAGTTG	
*ACT*	ACT-512F	ATGTGCAAGGCCGGTTTCG	Carbone and Kohn (1999)
	ACT-783 R	TACGAGTCCTTCTGGCCCAT	
*HIS3*	CYLH3F	AGGTCCACTGGTGGCAAG	Crous et al. (2004)
	CYLH3R	AGCTGGATGTCCTTGGACTG	

PCR was performed on the T100™ Thermal Cycler (Bio-Rad Laboratories Inc., CA, USA) in a 25-μL reaction volume containing 1.6 μl of dNTPs (2.5 mM μL^−1^ each), 0.2 μl of *Taq* polymerase (5 U μL^−1^), 2 μl of polymerase buffer (10× μL^−1^; Takara, Japan), 1 μl of each primer (25 mM μL^−1^) and 1 μl of genomic DNA (50 ng μL^−1^). The following conditions were used for the amplification of all genomic regions (except the ITS region): initial denaturation at 95°C for 5 min, followed by 32 cycles of denaturation at 94°C, annealing at 55°C, and extension at 72°C for 30 s each and a final extension at 72°C for 10 min. The ITS region was amplified under the following conditions: 94°C for 5 min, followed by 30 cycles at 94°C, 52°C and 72°C for 30 s each and lastly 72°C for 10 min. PCR products were sequenced by Sangon Biotech Co., Ltd. (Shanghai, China) using the same PCR primers as those used for PCR amplification.

### Phylogenetic analyses

Sequences of each gene or genomic region generated using forward and reverse primers were assembled with BioEdit v.7.2.5 (Hall, 1999). Then, consensus sequences were then combined with related sequences downloaded from GenBank, and aligned separately using Mafft v7.187 (Katoh and Standley, 2013) or manually when necessary. The nucleotide substitution model for each gene or genomic region was determined based on the Bayesian information criterion (BIC) using jModelTest v2.1.6 (Darriba et al., 2012). Phylogenetic trees based on ITS, *ACT*, *CHS-1*, *GAPDH*, *TUB2,* and *HIS3* datasets as well as a concatenated dataset were constructed using maximum likelihood (ML) and Bayesian inference (BI) analyses at the CIPRES web portal (Miller et al., 2010). The ML analysis was performed using the RAxML-HPC BlackBox tool (Stamatakis, 2014). The Markov Chain Monte Carlo (MCMC) algorithm for BI with two parallel runs of four chains was performed using MrBayes on XSEDE (Ronquist et al., 2012). Trees were sampled every 100 generations, and runs were stopped automatically when the average standard deviation of split frequencies fell below 0.01. A 50% majority rule consensus tree was summarized after discarding the first 25% samples. The resulting trees were visualized in FigTree v1.4.3 (Rambaut, 2016). In this study, 20 isolates were selected for phylogenetic analysis.

### Pathogenicity assays

To conform the Koch’s postulates, three *C. godetiae* isolates (Q-1, Q-2, and Q-3) were chosen for pathogenicity testing. Healthy sweet cherry fruits were selected in the field and sprayed with conidial suspensions (1 × 10^6^ conidia ml^−1^) or sterile distilled water (control). Five replicates of fruit were used for each conidial suspension. Disease development on the inoculated fruit was observed daily, and lesions on fruit were photographed at 5 days post-inoculation. The fungus was re-isolated from infected fruit after the appearance of symptoms. The morphological characteristics of these fungal isolates were compared with those originally used as inoculum.

### Fungicide sensitivity of isolates

Referring to the fungicides with high efficiency and low intensity used for the control of other plant anthracnose, 13 kinds of fungicides were selected for preliminary screening of Q-1 isolates (Table 2). Thirteen fungicides, including difenoconazole, propiconazole, prochloraz-manganese, azoxystrobin, pyraclostrobin, chlorothalonil, dithianon, polyantimycin, zhongshengmycin, trifloxystrobin-tebuconazole, difenoconazole-azoxystrobin, bromothalonil, and polysaccharide, were dissolved in sterile water to prepare 10 mg/ml stock solutions. Half maximal effective concentration (EC_50_) values indicating fungicide sensitivity were determined using mycelial growth assays. The PDA medium plates, containing a series of final concentrations of each fungicide (Table 3), were prepared by adding an appropriate volume of the stock solution (in sterile water). The margin of 6-day-old colonies cultured on PDA was used to generate 5-mm mycelial plugs, which were placed at the center of PDA plates containing different concentrations of fungicides. All plates were incubated at 25°C for 6 days. The growth inhibition rate of mycelia was calculated by the following formula: *i* = (a1 − a2)/a1 × 100, where *i* is the growth inhibition rate of mycelia, a1 is the hyphae area of untreated pathogen, and a2 is the hyphae area of treated pathogen (Etebarian et al., 2005). The EC50 (concentration for 50% of maximal effect) values of different plant extracts were calculated using IBM SPSS analytics (SPSS Inc., Chicago, IL, United States) (Mo et al., 2021). Each fungicide treatment and control contained three replicate plates, and the experiment was performed twice.

**Table 2:** Information on 13 fungicides.

Name	Registration code	Holder of registration certificate
Difenoconazole	PD20121392	Shanxi Yitianfeng Crop Technology Co., Ltd.
Propiconazole	PD20093418	Shandong Biannong Sida Biotechnology Co., Ltd.
Prochloraz-manganese	PD20151437	Jiangsu Suzhou Fumishi Plant Protection Agent Co., Ltd.
Pyraclostrobin	PD20180392	Shijiazhuang Huaxing Pesticide Co., Ltd.
Azoxystrobin	PD20142114	Syngenta Nantong Crop Protection Co., Ltd.
Trifloxystrobin-tebuconazole	PD20180677	Jiangsu Jiannong Plant Protection Co., Ltd.
Difenoconazole-azoxystrobin	PD20150707	Syngenta Nantong Crop Protection Co., Ltd.
Chlorothalonil	PD86180-5	Limin Chemical Co., Ltd.
dithianon	PD20096835	Jiangxi Heyi Chemical Co., Ltd.
Polyantimycin	PD20182377	Shandong Rushan Hanwei Biotechnology Co., Ltd.
Zhongshengmycin	PD20182317	Hebei Zhongbaolv Crop Technology Co., Ltd.
Bromothalonil	PD20094687	Jiangsu Tuoqiu Agrochemical Co., Ltd.
Polysaccharides	PD20142390	Liaoning Weike Biological Engineering Co., Ltd.

**Table 3:** The sensitivities of *Colletotrichum godetiae* isolate Q-1 to 13 fungicides.

Clades	Fungicides	Concentration (μg mL^−1^)					EC_50_ (μg mL^−1^)	Correlation coefficient
		C1	C2	C3	C4	C5		
DMIs	Difenoconazole	0.001	0.01	0.1	1	10	0.15	0.9948
	Propiconazole	0.15	0.44	1.33	4	12	0.26	0.9935
	Prochloraz-manganese	0.001	0	0.01	0.03	0.1	0.04	0.9642
QoIs	Pyraclostrobin	0.16	0.31	0.63	1.25	2.5	0.14	0.9912
	Azoxystrobin	0.05	0.5	5	50	500	1.08	0.9768
Compounds of DMI and QoI	Trifloxystrobin-tebuconazole	0.005	0.022	0.087	0.35	1.39	0.08	0.9923
	Difenoconazole-azoxystrobin	0.002	0.007	0.02	0.07	0.2	0.1	0.9948
Glycolysis inhibitors	Chlorothalonil	0.62	1.85	5.56	16.67	50	91.26	0.9927
	dithianon	0.12	1.2	12	120	1,200	10.27	0.9855
Antibiotics	Polyantimycin	1.25	5	20	80	320	21.49	0.9463
	Zhongshengmycin	0.19	0.96	4.8	24	120	4.07	0.9812
Bromothalonil	Bromothalonil	0.44	1.33	4	12	36	15.29	0.9869
Polysaccharides	Polysaccharides	3.91	15.63	62.5	250	1,000	22.54	0.9555

A suitable fungicide was selected based on the preliminary screening of 13 fungicides using the Q-1 isolate. Then, the sensitivity of 20 isolates (5, 5, 2, and 8 isolates from Bijie, Guiyang, Liupanshui, and Qianxinanzhou, respectively) to the suitable fungicide was tested as described above.

## Results

### Disease symptoms and fungal isolation

**Figure 1:**
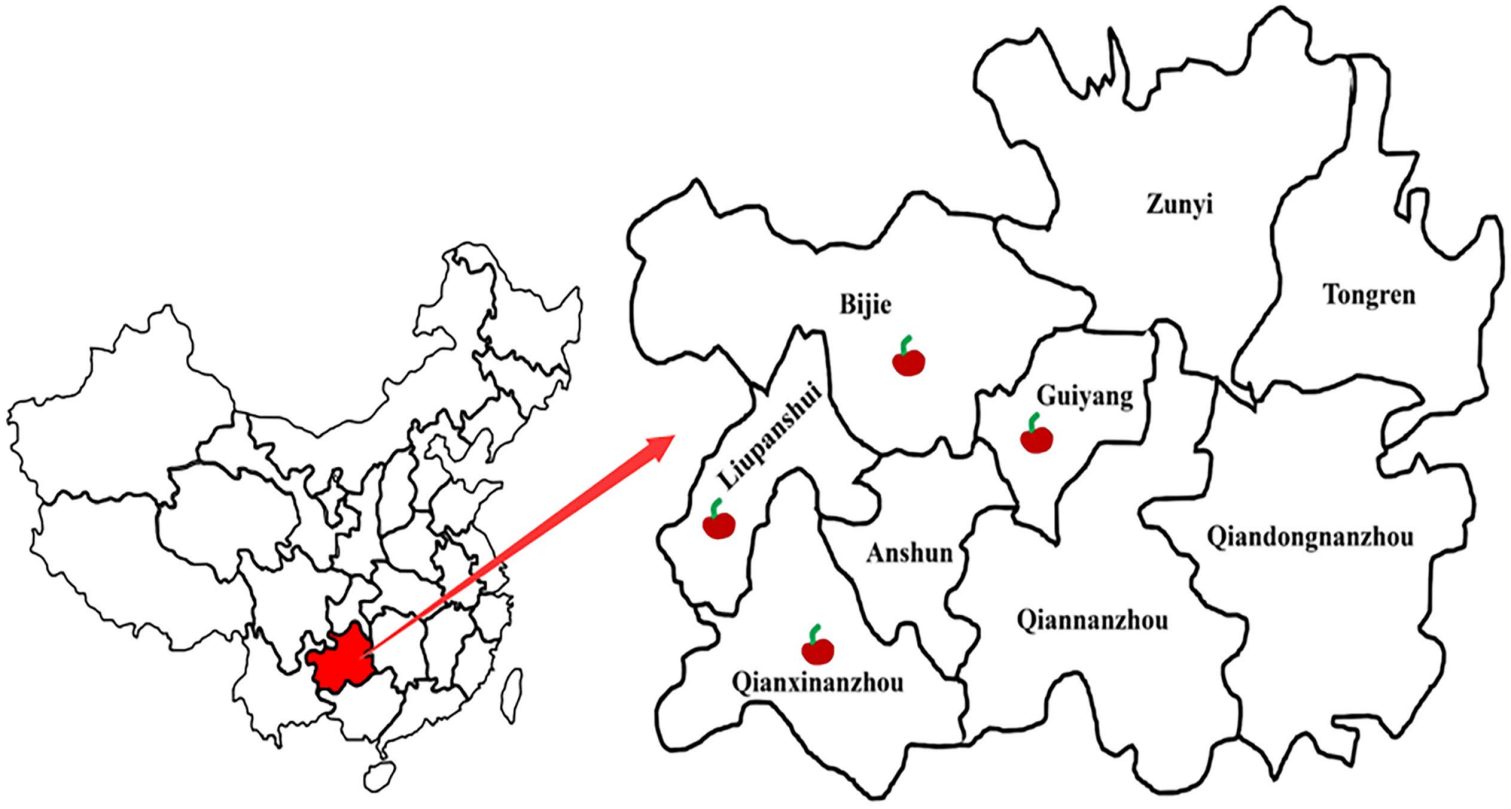
Distribution of cherry sample sites in four regions in Guizhou, China.

**Figure 2:**
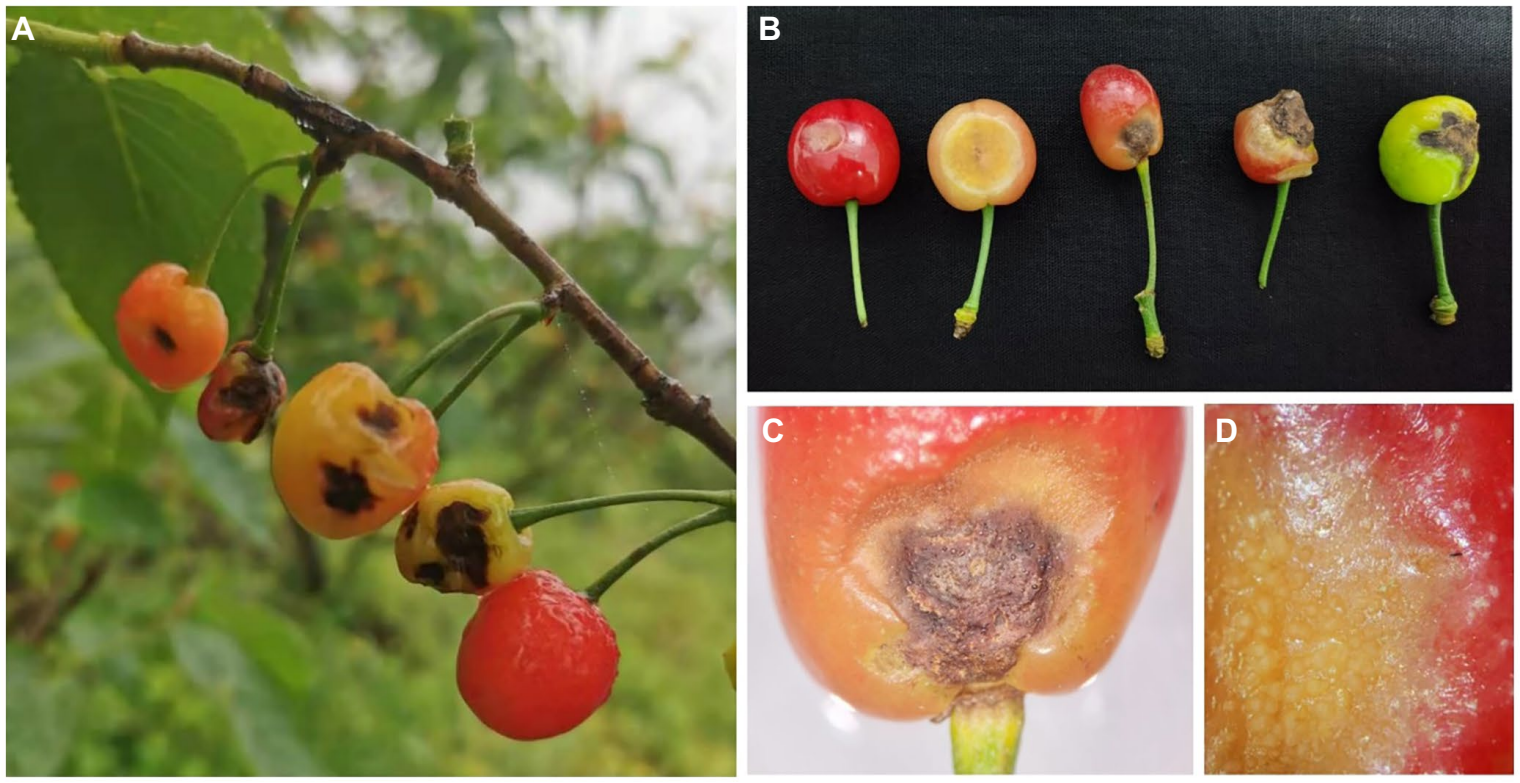
The symptoms of cherry anthracnose on fruits. **(A)** Symptoms on fruits in field. **(B)** Symptoms on fruits at different development stages. **(C)** Necrotic spot on mature fruit. **(D)** Margin of lesions on mature fruit.

**Figure 3:**
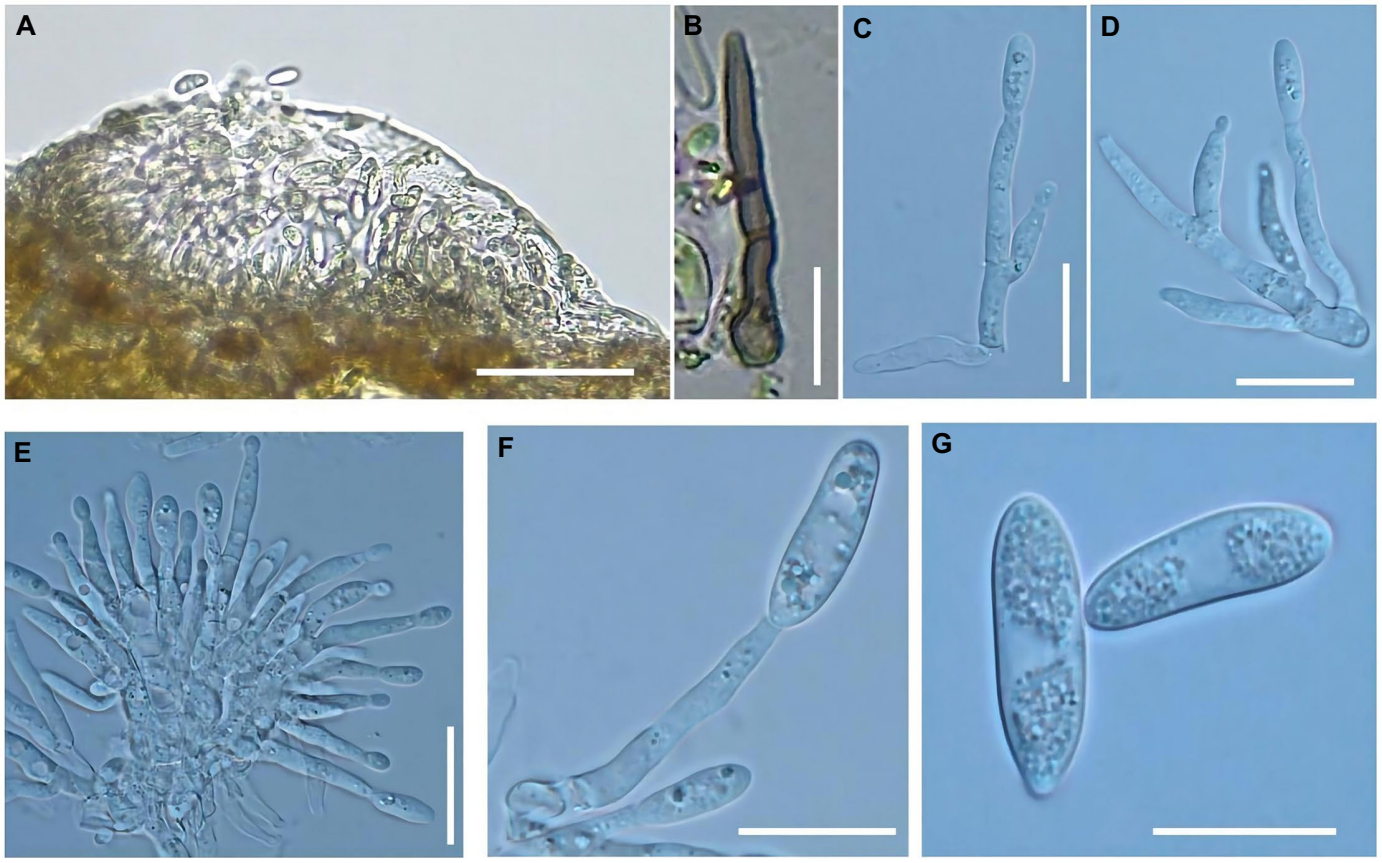
Morphological characteristics of *Colletotrichum godetiae* were observed on the diseased fruits. **(A)** Acervuli, bar = 50 μm. **(B)** Setae, bar = 20 μm. **(C–F)** Conidiophores, bar = 10 μm. **(G)** Conidia, bar = 10 μm.

A fruit disease emerged in four major ‘Manaohong’ cherry production areas (Bijie, Guiyang, Liupanshui, and Qianxinanzhou) in Guizhou province (Figure 1). Symptomatic sweet cherry fruits displaying lesion collapsed but maintained an intact peel. At the early stage of infection, yellow, caviar-like patches were formed on the fruit surface. With increased disease severity over time, the fruit became rotten, and lesions turned black (Figures 2A–D). Microscopic analysis revealed the presence of brown acervuli (Figure 3A) occasionally branched hyaline conidiophores (Figures 3C–F), black setae (Figure 3B), and aseptate, cylindrical, and slightly curved conidia (Figure 3G) on the surface of diseased fruits, suggesting that the disease was caused by *Colletotrichum* species. To confirm the identity of the causal agent, a total of 116 *Colletotrichum* isolates were isolated from diseased sweet cherry fruits using the single-spore separation method. We classified the obtained 116 isolates according to the collection location and colony morphology. We divided these strains into three categories according to the colony morphology, and finally selected 20 strains for the next experiment. In these 20 isolates, 5 (named as B-1 to B-5), 5 (G-1 to G-5), 2 (L-1 to L-2), and 8 (Q-1 to Q-8) *Colletotrichum* isolates, obtained from Bijie, Guiyang, Liupanshui and Qianxinanzhou, respectively, were chosen for further study.

### Morphological characteristics

**Figure 4:**
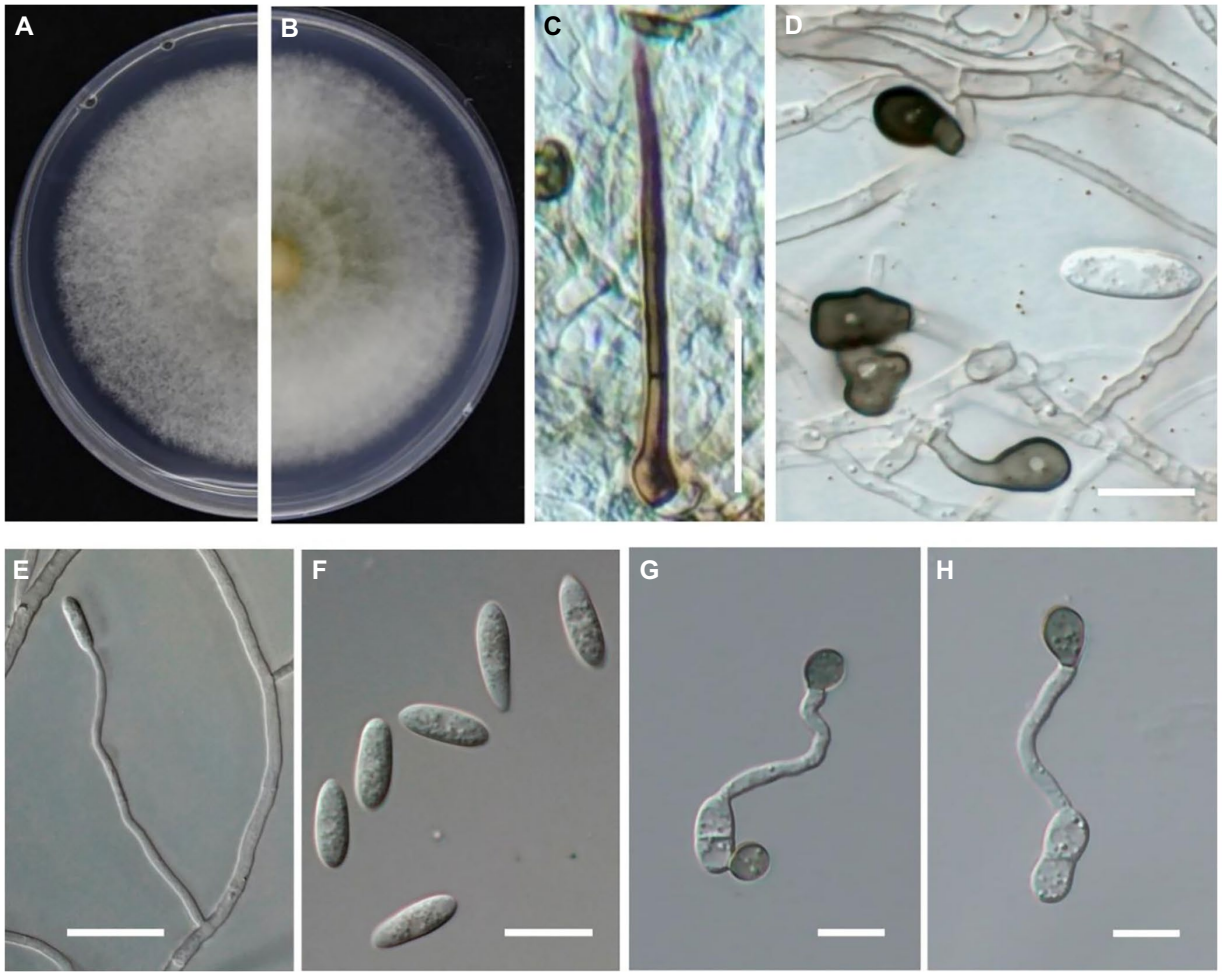
Morphological characteristics of *Colletotrichum godetiae* cultured on PDA plates. **(A,B)** Colony morphology on upside **(A)** and underside **(B)** of *C. godetiae* on PDA after 7 days at 25°C. **(C)** Setae, bar = 20 μm. **(D)** Mycelial appressorium, bar = 10 μm. **(E)** Conidiophores, bar = 10 μm. **(F)** Conidia, bar = 10 μm. **(G,H)** Appressorium, bar = 10 μm.

After growth on PDA at 25°C for 6 days, the colonies were light gray at the margin, dark gray at the center and the underside, with dense and cottony aerial mycelium (Figures 4A,B). Its growth rate was 6.6 mm per day, and its growth rate was consistent with each repetition. Setae were dark brown and acicular (Figure 4C). The mycelial appressorium was dark brown or black in color and elliptical or irregular in shape (Figure 4D), and ranged from 6.9–11.1 × 4.2–7.4 μm in size (*n* = 30). Conidiophores were hyaline, smooth-walled, and crooked with no branches (Figure 4E). Conidia on PDA were transparent with aseptate, cylindrical, and slightly curved (Figure 4F), and ranged in size from 12.5–17.6 × 3.5–5.3 μm (*n* = 40). The appressoria were dark brown or black and elliptical or irregular (Figures 4G,H), and ranged in size from 7.7–10.8 × 6.2–9.8 μm (*n* = 30). The color and shape of setae, conidiophores, and conidia formed on PDA plates and on diseased fruit were similar. Overall, the morphological characteristics of the isolates determined in this study were consistent with those of *C. godetiae* described by Damm et al. (2012).

### Phylogenetic analysis

**Figure 5:**
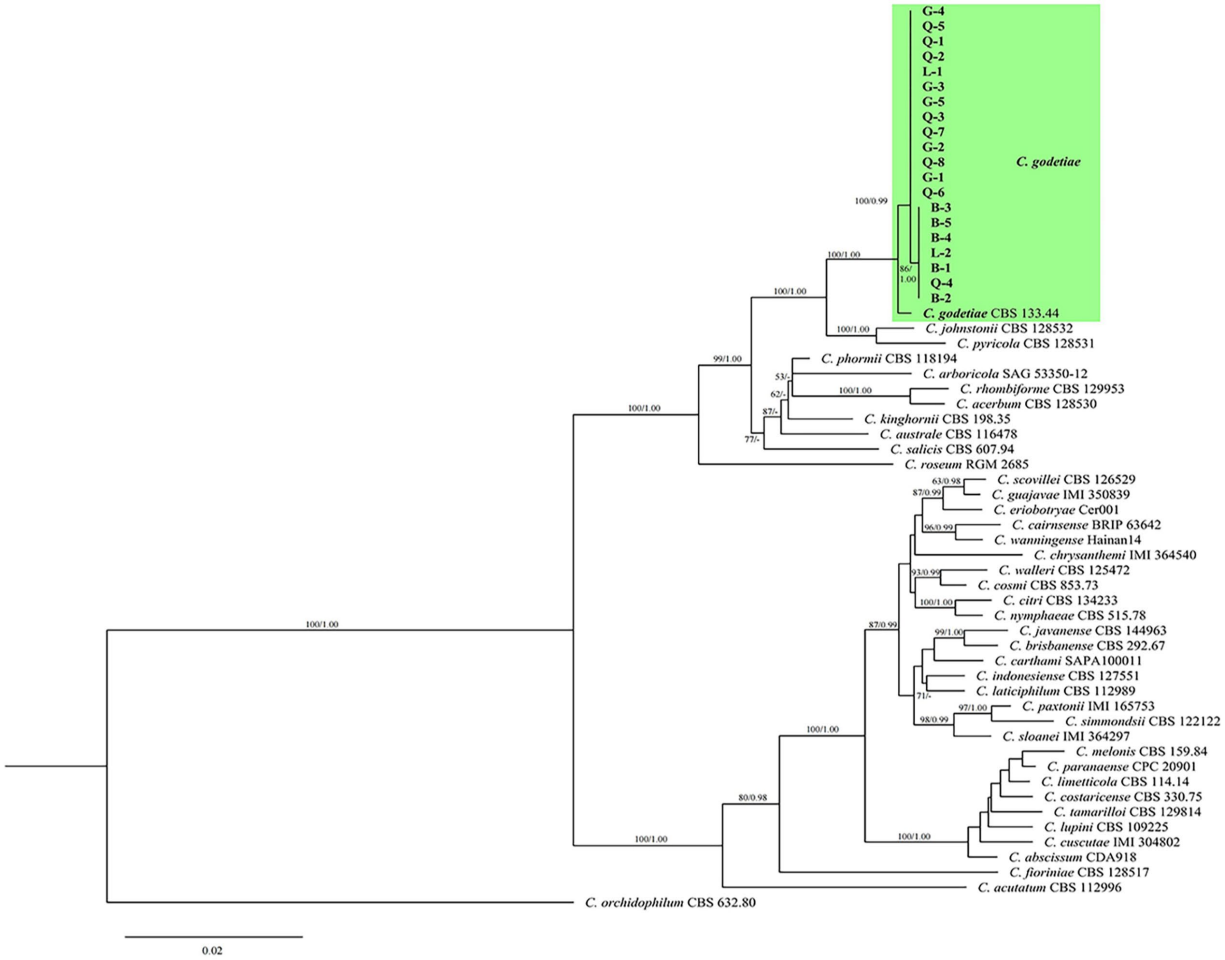
Maximum-likelihood (ML) and Bayesian inference (BI) phylogenetic tree illustrating the relationships with the godetiae species complex and the *Colletotrichum* strains isolated from diseased cherry fruits in Guizhou. Bootstrap support values for ML greater than 50% and Bayesian posterior probabilities greater than 0.90 are shown next to topological nodes.

Twenty c*olletotrichum* isolates (B-1 to B-5, G-1 to G-5, L-1 to L-2, and Q-1 to Q-8) obtained from the four cities were selected for further analysis. Six DNA fragments (ITS, *ACT*, *CHS-1*, *GAPDH*, *TUB2,* and *HIS3*) combined a gene alignment data matrix was used to perform phylogenetic analysis. The sequences of Six PCR fragments of each isolate were deposited in GenBank, and the accession numbers are listed in Table 4. In Figure 5, the results showed that our new collections (20 isolates) were clustered with *C. godetiae*. Based on the multilocus phylogenetic analyses of five genomic regions and morphological characteristics of colonies, conidia, appressoria, conidiophores, and setae, the isolates were identified as *C. godetiae*.

**Table 4:** Information of *Colletotrichum* spp. used in this study for phylogenetic analyses.

Taxon	Strains	GenBank accession number					
		ITS	*GAPDH*	*CHS-1*	*ACT*	*TUB2*	*HIS3*
*C. abscissum*	CDA 918	KP843126	KP843129	KP843132	KP843141	KP843135	KP843138
*C. acerbum*	CBS 128530	JQ948459	JQ948790	JQ949120	JQ949780	JQ950110	JQ949450
*C. acutatum*	CBS 112996	JQ005776	JQ948677	JQ005797	JQ005839	JQ005860	JQ005818
*C. arboricola*	SAG 53350–12	MH817944	MH817950	—	MH817956	MH817962	—
*C. australe*	CBS 116478	JQ948455	JQ948786	JQ949116	JQ949776	JQ950106	JQ949446
*C. brisbanense*	CBS 292.67	JQ948291	JQ948621	JQ948952	JQ949612	JQ949942	JQ949282
*C. cairnsense*	BRIP 63642	KU923672	KU923704	KU923710	KU923716	KU923688	KU923722
*C. carthami*	SAPA100011	AB696998	—	—	—	AB696992	—
*C. chrysanthemi*	IMI 364540	JQ948273	JQ948603	JQ948934	JQ949594	JQ949924	JQ949264
*C. citri*	CBS 134233	KC293581	KC293741	KY856138	KY855973	KC293661	KY856309
*C. cosmi*	CBS 853.73	JQ948274	JQ948604	JQ948935	JQ949595	JQ949925	JQ949265
*C. costaricense*	CBS 330.75	JQ948180	JQ948510	JQ948841	JQ949501	JQ949831	JQ949171
*C. cuscutae*	IMI 304802	JQ948195	JQ948525	JQ948856	JQ949516	JQ949846	JQ949516
*C. eriobotryae*	Cer 001	MF772487	MF795423	MN191653	MN191648	MF795428	MN191658
*C. fioriniae*	CBS 128517	JQ948292	JQ948622	JQ948953	JQ949613	JQ949943	JQ949283
***C. godetiae***	**Q-1**	OK336098	ON241073	ON241053	ON241033	ON241113	ON241093
***C. godetiae***	**Q-2**	OK336099	ON241074	ON241054	ON241034	ON241114	ON241094
***C. godetiae***	**Q-3**	OK336100	ON241075	ON241055	ON241035	ON241115	ON241095
***C. godetiae***	**Q-4**	OK336101	ON241076	ON241056	ON241036	ON241116	ON241096
***C. godetiae***	**Q-5**	OK336102	ON241077	ON241057	ON241037	ON241117	ON241097
***C. godetiae***	**Q-6**	OK336103	ON241078	ON241058	ON241038	ON241118	ON241098
***C. godetiae***	**Q-7**	OK336104	ON241079	ON241059	ON241039	ON241119	ON241099
***C. godetiae***	**Q-8**	OK336105	ON241080	ON241060	ON241040	ON241120	ON241100
***C. godetiae***	**G-1**	OK336106	ON241081	ON241061	ON241041	ON241121	ON241101
***C. godetiae***	**G-2**	OK336107	ON241082	ON241062	ON241042	ON241122	ON241102
***C. godetiae***	**G-3**	OK336108	ON241083	ON241063	ON241043	ON241123	ON241103
***C. godetiae***	**G-4**	OK336109	ON241084	ON241064	ON241044	ON241124	ON241104
***C. godetiae***	**G-5**	OK336110	ON241085	ON241065	ON241045	ON241125	ON241105
***C. godetiae***	**B-1**	OK336111	ON241086	ON241066	ON241046	ON241126	ON241106
***C. godetiae***	**B-2**	OK336112	ON241087	ON241067	ON241047	ON241127	ON241107
***C. godetiae***	**B-3**	OK336113	ON241088	ON241068	ON241048	ON241128	ON241108
***C. godetiae***	**B-4**	OK336114	ON241089	ON241069	ON241049	ON241129	ON241109
***C. godetiae***	**B-5**	OK336115	ON241090	ON241070	ON241050	ON241130	ON241110
***C. godetiae***	**L-1**	OK336116	ON241091	ON241071	ON241051	ON241131	ON241111
***C. godetiae***	**L-2**	OK336117	ON241092	ON241072	ON241052	ON241132	ON241112
*C. godetiae*	CBS 133.44	JQ948402	JQ948733	JQ949063	JQ949723	JQ950053	JQ949393
*C. godetiae*	CPO 27.921	MN744275	MN737334	MN746542	MN746509	MN848358	MN848382
*C. guajavae*	IMI 350839	JQ948270	JQ948600	JQ948931	JQ949591	JQ949921	JQ949261
*C. indonesiense*	CBS 127551	JQ948288	JQ948618	JQ948949	JQ949609	JQ949939	JQ949279
*C. javanense*	CBS 144963	MH846576	MH846572	MH846573	MH846575	MH846574	MH846571
*C. johnstonii*	CBS 128532	JQ948444	JQ948775	JQ949105	JQ949765	JQ950095	JQ949435
*C. kinghornii*	CBS 198.35	JQ948454	JQ948785	JQ949115	JQ949775	JQ950105	JQ950105
*C. laticiphilum*	CBS 112989	JQ948289	JQ948619	JQ948950	JQ949610	JQ949940	JQ949280
*C. lauri*	IT2505-1a	KY514347	KY514344	KY514341	KY514338	KY514350	—
*C. limetticola*	CBS 114.14	JQ948193	JQ948523	JQ948854	JQ949514	JQ949844	JQ949184
*C. lupini*	CBS 109225	JQ948155	JQ948485	JQ948816	JQ949476	JQ949806	JQ949146
*C. melonis*	CBS 159.84	JQ948194	JQ948524	JQ948855	JQ949515	JQ949845	JQ949185
*C. nymphaeae*	CBS 515.78	JQ948197	JQ948527	JQ948858	JQ949518	JQ949848	JQ949188
*C. paranaense*	CPC 20901	KC204992	KC205026	KC205043	KC205077	KC205060	KC205004
*C. paxtonii*	IMI 165753	JQ948285	JQ948615	JQ948946	JQ949606	JQ949936	JQ949276
*C. phormii*	CBS 118194	JQ948446	JQ948777	JQ949107	JQ949767	JQ950097	JQ949437
*C. pyricola*	CBS 128531	JQ948445	JQ948776	JQ949106	JQ949766	JQ950096	JQ949436
*C. rhombiforme*	CBS 129953	JQ948457	JQ948788	JQ949118	JQ949778	JQ950108	JQ949448
*C. roseum*	RGM 2685	MK903611	MK903603	—	MK903604	MK903607	—
*C. salicis*	CBS 607.94	JQ948460	JQ948791	JQ949121	JQ949781	JQ950111	JQ949451
*C. scovillei*	CBS 126529	JQ948267	JQ948597	JQ948928	JQ949588	JQ949918	JQ949258
*C. simmondsii*	CBS 122122	JQ948276	JQ948606	JQ948937	JQ949597	JQ949927	JQ949267
*C. sloanei*	IMI 364297	JQ948287	JQ948617	JQ948948	JQ949608	JQ949938	JQ949278
*C. tamarilloi*	CBS 129814	JQ948184	JQ948514	JQ948845	JQ949505	JQ949835	JQ949175
*C. walleri*	CBS 125472	JQ948275	JQ948605	JQ948936	JQ949596	JQ949926	JQ949266
*C. wanningense*	Hainan14	MG830462	MG830318	MG830302	MG830270	MG830286	—
*C. orchidophilum*	CBS 632.80	JQ948151	JQ948481	JQ948812	JQ949472	JQ949802	JQ949142

*The C. godetiae isolates obtained in this study are in bold*.

### Pathogenicity test

**Figure 6:**
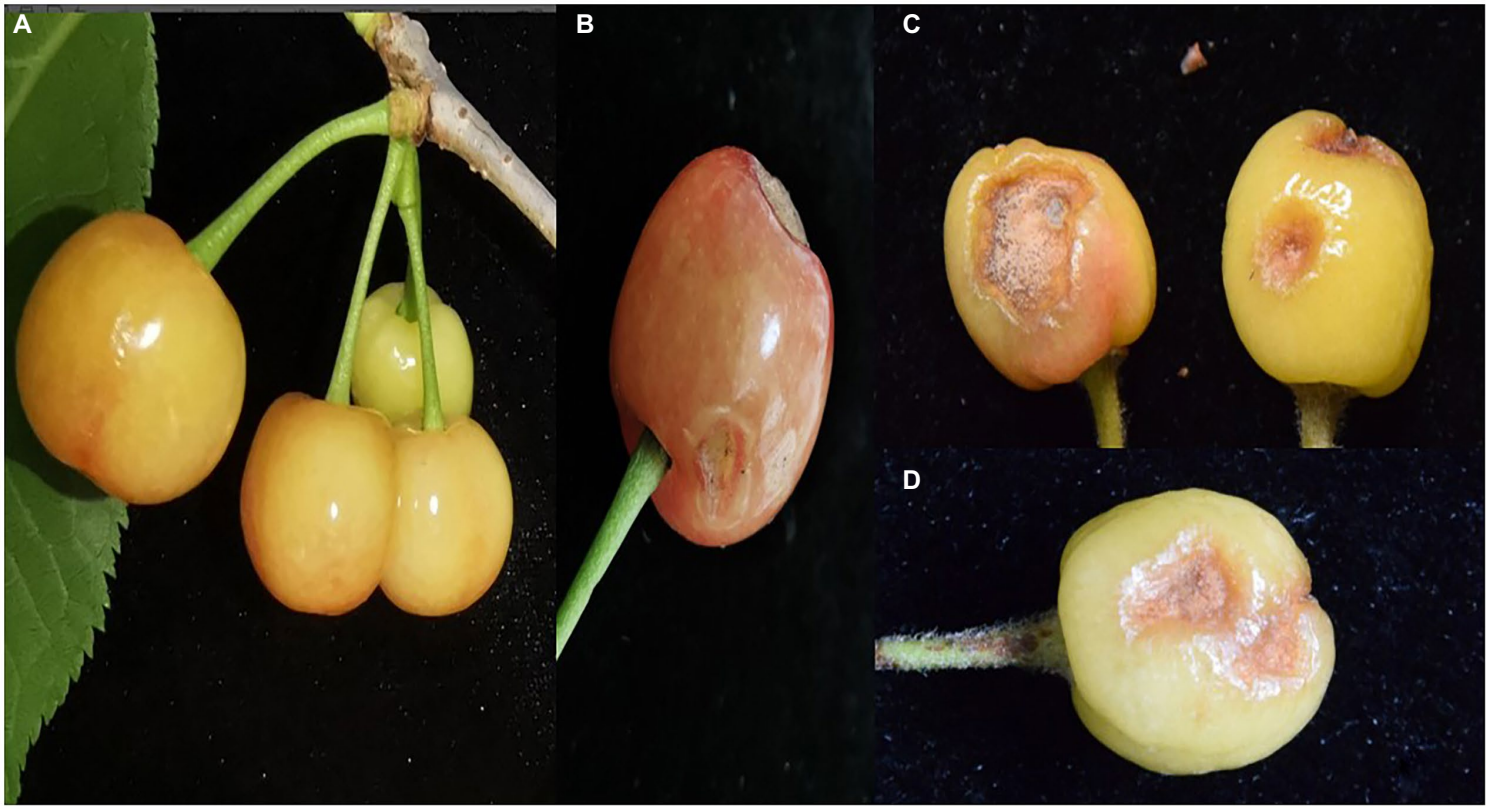
Pathogenicity test of *Colletotrichum godetiae* isolates obtained from Qianxinanzhou. **(A)** CK was treated with sterilized distilled water. **(B–D)** Lesions on cherry fruits were inoculated with Q-1, Q-2 and Q-3 isolates, respectively.

Pathogenicity tests were performed to satisfy Koch’s postulates by artificially inoculating sweet cherry fruits with spore suspensions of three isolates (Q-1, Q-2, and Q-3) of *C. godetiae*. After 5 days, all *C. godetiae* inoculated fruits exhibited necrotic lesions with yellowish colonies (Figures 6B–D), similar to the symptoms initially observed on naturally infected fruits. Fruits inoculated with distilled water were asymptomatic (Figure 6A). The morphological characteristics of the fungal pathogen re-isolated from the inoculated fruits were identical to those of the *C. godetiae* strains originally obtained from sweet cherry fruits. Therefore, *C. godetiae* was confirmed as the causal agent of anthracnose on sweet cherry fruits.

### Fungicide sensitivity testing

To identify fungicides effective in controlling the anthracnose disease, 13 fungicides were divided into seven classes (DMIs, QoIs, glycolysis inhibitors, antibiotics, compounds of DMI and QoI, bromothalonil, and polysaccharides) were used in this study. First, the Q-1 isolate of *C. godetiae* was selected to perform the sensitivity assay. The 13 fungicides all had different degrees of inhibitory effect on the growth of C. godetiae, and the inhibitory effect gradually increased with the increase of chemical concentration. The results in Table 3 show that among the different control agents, prochloraz-manganese have the best inhibitory effects, EC_50_ were 0.04 μg ml^−1^, respectively, of which the inhibitory effect of trifloxystrobin-tebuconazole and difenoconazole-azoxystrobin followed, with EC_50_ values of 0.08 and 0.10 μg ml^−1^, and the worst inhibitory effect was chlorothalonil, whose EC_50_ was 91.26 μg ml^−1^.

We selected 10 fungicides with better inhibitory effect among 13 fungicides and carried out extensive inhibition tests, and found that six fungicides in these 10 fungicides showed high sensitivity to the 20 isolates (B-1 to B-5, G-1 to G-5, L-1 to L-2, Q-1 to Q-8) of *C. godetiae*, and no drug-resistant groups appeared. The six fungicides are DMIS fungicide (difenoconazole, propiconazole, and prochloraz-manganese), QOIS fungicide (pyraclostrobin), and DMI and QoI fungicide compounds (trifloxystrobin-tebuconazole and difenoconazole-azoxystrobin), and their EC50 values ranged from 0.013 to 1.563 μg ml^−1^ (Table 5). These results suggest that these five fungicides could be used for controlling *C. godetiae*.

**Table 5:** The sensitivity determination of 20 *Colletotrichum godetiae* isolates to 10 fungicides.

Isolates	EC50 (μg mL-1)									
	Difenoconazole	Propiconazole	Prochloraz-manganese	Pyraclostrobin	Azoxystrobin	Trifloxystrobin-tebuconazole	Difenoconazole-azoxystrobin	dithianon	Zhongshengmycin	Bromothalonil
*C. godetiae Q-1*	0.14	0.26	0.04	0.14	1.08	0.15	0.10	10.27	4.07	15.29
*C. godetiae Q-2*	0.12	0.67	0.09	0.12	0.91	0.15	0.10	50.41	6.42	29.32
*C. godetiae Q-3*	0.14	0.83	0.13	0.14	0.47	0.28	0.45	33.64	7.52	45.17
*C. godetiae Q-4*	0.20	0.70	0.08	0.20	0.20	0.20	0.23	34.37	10.13	46.16
*C. godetiae Q-5*	0.11	0.95	0.35	0.11	5.12	0.13	0.79	18.52	8.63	50.16
*C. godetiae Q-6*	0.14	1.01	0.11	0.14	1.66	0.11	0.23	23.50	7.29	52.67
*C. godetiae Q-7*	0.15	0.71	0.13	0.15	17.25	0.12	0.48	15.30	10.38	53.98
*C. godetiae Q-8*	0.13	0.97	0.13	0.13	10.16	0.18	0.55	20.56	10.95	60.47
*C. godetiae G-1*	0.17	0.99	0.12	0.17	2.15	0.24	1.33	19.49	7.83	61.56
*C. godetiae G-2*	0.14	0.41	0.10	0.44	72.31	0.13	1.00	53.02	6.28	66.96
*C. godetiae G-3*	0.12	0.58	0.12	0.12	1.55	0.15	0.85	21.40	10.31	67.90
*C. godetiae G-4*	0.11	0.73	0.07	0.11	7.24	0.28	1.43	15.59	4.57	68.08
*C. godetiae G-5*	0.06	1.06	0.15	0.06	3.14	0.19	1.01	11.80	15.65	99.02
*C. godetiae B-1*	0.02	0.22	0.07	0.02	3.41	0.22	1.56	16.29	5.79	72.89
*C. godetiae B-2*	0.03	0.48	0.14	0.03	2.04	0.13	0.67	2.89	9.49	73.74
*C. godetiae B-3*	0.02	0.42	0.06	0.02	24.35	0.19	0.36	54.62	13.53	83.18
*C. godetiae B-4*	0.05	0.46	0.11	0.05	1.42	0.14	0.11	18.91	2.85	10.87
*C. godetiae B-5*	0.01	1.02	0.17	0.01	0.70	0.18	0.38	34.22	5.10	13.79
*C. godetiae L-1*	0.04	1.19	0.11	0.04	4.12	0.13	1.00	27.62	7.34	67.90
*C. godetiae L-2*	0.14	1.03	0.17	0.14	0.14	0.25	0.54	23.52	33.39	66.96

## Discussion

*Colletotrichum* species cause anthracnose disease of the leaf, young shoot, and especially fruit of sweet cherry trees, resulting in great economic losses (Dean et al., 2012). Recently, sweet Cherry fruit anthracnose has occurred in a large area in Guizhou province of China, and the disease has become a major factor restricting the development of this industry. However, the cause of the disease is still unclear, and there is no targeted prevention and treatment method. To determine the cause of this problem, 116 isolates of *Colletotrichum* species were isolated from four Guizhou cities with high anthracnose incidence. Based on morphological characteristics (colony color, mycelial growth rate, and shape and size of the mycelial appressorium, conidia, and appressorium) and molecular data (sequences of ITS, *ACT*, *CHS-1*, *GAPDH*, *TUB2,* and *HIS3*), the causal agent was identified as *C. godetiae* (belonging to the *acutatum* species complex) (Damm et al., 2012). This is also the first report showing that *C. godetiae* (belonging to the acutatum species complex) is responsible for causing sweet cherry anthracnose in China.

*C. godetiae* was originally isolated from the seeds of *Clarkia* (syn. *Godetia*) (Damm et al., 2012). Currently, *C. godetiae* has a number of hosts worldwide, including plant species belonging to the Adoxaceae, Anacardiaceae, Berberidaceae, Fabaceae, Juglandaceae, Myrtaceae, Oleaceae, Onagraceae, Podocarpaceae, Rosaceae, Rhamnaceae, Rutaceae, Solanaceae, and Vitaceae families, resulting in leaf spots, fruit rot, die back and stem end rot (Afanador-Kafuri et al., 2014; Baroncelli et al., 2014, 2015, 2017; Mosca et al., 2014; Munda, 2014; Talhinhas et al., 2015; Wang et al., 2017, 2019, 2020; Zhang et al., 2020; Shi et al., 2021). Recently, a study on walnut anthracnose identified *C. fioriniae* and *C. godetiae* in nuts and buds in the same orchard (Da Lio et al., 2018), suggesting that the pathogen population responsible for walnut anthracnose was complex. Therefore, distinguishing among the different *Colletotrichum* species is particularly important for accurate pathogen identification. In this study, we obtained 116 *Colletotrichum* isolates from 40 diseased sweet cherry fruits collected from four regions in Guizhou. The morphological characteristics of these isolates, including colony color, the shape, and size of mycelial appressorium, conidia, and appressorium, were similar to those of *C. godetiae* (Damm et al., 2012). To fulfill Koch’s postulates, spore suspensions of three isolates of *C. godetiae* (Q-1, Q-2, and Q-3) were sprayed on sweet cherry fruits in this study. The inoculation results showed that all isolates could infect sweet cherry fruits, causing yellow and sunken lesions, which was consistent with the naturally infected sweet cherry fruit samples. The morphological and molecular data of the re-isolated isolates confirmed that *C. godetiae* is associated with sweet cherry anthracnose in Guizhou province.

No crop completely immune to the various isolates of *Colletotrichum* species has been reported to date (Dean et al., 2012). As a result, chemical control is still considered as the most effective and important management strategy for controlling the anthracnose disease. At present, QoIs and DMIs are the major groups of fungicides used to control anthracnose in agricultural crops worldwide (Li et al., 2005; Ji et al., 2014; Hu et al., 2015; Forcelini et al., 2016; Yokosawa et al., 2017; Baggio et al., 2018; Wang et al., 2019; Kongtragoul et al., 2020; Shi et al., 2020). However, several reports have described a few QoI (azoxystrobin) resistant isolates belonging to the C. *gloeosporioides* species complex, such as *C. gloeosporioides* (Inada et al., 2008; Kim et al., 2016), *C. siamense* (Hu et al., 2015; Zhang et al., 2020) and *C. fructicola* (Yokosawa et al., 2017; Zhang et al., 2020), indicating that these fungicides may not be effective in controlling the anthracnose disease in some areas. Therefore, to identify suitable fungicides for controlling sweet cherry anthracnose-causing *C. godetiae*, 13 fungicides with different modes of action were evaluated in this study. Our results showed that the EC_50_ values of antibiotics (polyantimycin and zhongshengmycin), glycolysis inhibitors (chlorothalonil and dithianon), bromothalonil, and polysaccharide fungicides effective against the Q-1 isolate ranged from 4.07 to 91.26 μg ml^−1^, while those of DMIs (prochloraz-manganese, difenoconazole, and propiconazole), QoIs (azoxystrobin and pyraclostrobin), compounds of DMI and QoI (trifloxystrobin-tebuconazole and difenoconazole-azoxystrobin) ranged from 0.04 to 1.18 μg ml^−1^. These results indicated that the Q-1 isolate is quite sensitive to DMI, QoI and compound of DMI and QoI fungicides. At the same time, we selected 10 fungicides with better inhibitory effects for extensive inhibition tests on *C. godetiae* isolates, and found that these 20*\u00B0C. godetiae* isolates (B-1 to B-5, G-1 to G-5, L-1 to L-2, Q-1 to Q-8) showed strong sensitivity to 5 fungicides (difenoconazole, propiconazole, prochloraz-manganese, pyraclostrobin, trifloxystrobin-tebuconazole, and difenoconazole-azoxystrobin), and their EC50 values ranged from is 0.013 to 1.563 μg ml^−1^, suggesting that these fungicides would be ideal for controlling sweet cherry anthracnose in Guizhou, China. However, the effect of 6 fungicides on *C. godetiae* remains unknown under field conditions and needs further study.

In this study, we showed for the first time that *C. godetiae* causes anthracnose in sweet cherry in Guizhou province, China. *C. godetiae* was identified as the causal agent of sweet cherry anthracnose based on morphological characterization, phylogenetic analyses, and pathogenicity assays. Additionally, fungicide sensitivity assays showed that the isolates were highly sensitive to difenoconazole, propiconazole, prochloraz-manganese, pyraclostrobin, trifloxystrobin-tebuconazole, and difenoconazole-azoxystrobin. Overall, this study provides crucial information for the effective control of sweet cherry anthracnose in China.

## Data availability statement

The names of the repository/repositories and accession number(s) can be found below: https://www.ncbi.nlm.nih.gov/nuccore. The accession numbers of the sequences deposited in GenBank are: ITS: OK336098-OK336117; ACT: ON241033-ON241052; CHS-1: ON241053-ON241072; GAPDH: ON241073-ON241092; HIS3: ON241093-ON241112; TUB2: ON241113-ON241132.

## Author contributions

KP and YP conducted the experiments. TT and XZe analyzed the data. ML and SJ prepared the figures and tables. FT and ZZ designed the project and supervised the experiments. XZh drafted the manuscript. All authors contributed to the article and approved the submitted version.

## Funding

This work was supported by grants from the Guizhou Provincial Science and Technology Project, grant number Support of QKH [2021] General 199 and the National Natural Science Foundation of China (NSFC: 32000013).

## Conflict of interest

The authors declare that the research was conducted in the absence of any commercial or financial relationships that could be construed as a potential conflict of interest.

## Publisher’s note

All claims expressed in this article are solely those of the authors and do not necessarily represent those of their affiliated organizations, or those of the publisher, the editors and the reviewers. Any product that may be evaluated in this article, or claim that may be made by its manufacturer, is not guaranteed or endorsed by the publisher.

## References

[ref1] Afanador-Kafuri L. González A. Gañán L. Mejía J. F. Cardona N. Alvarez E. (2014). Characterization of the *Colletotrichum* species causing anthracnose in Andean blackberry in Colombia. Plant Dis. 98, 1503–1513. doi: 10.1094/pdis-07-13-0752-RE, PMID: 30699787

[ref2] Baggio J. S. Wang N. Y. Peres N. A. Amorim L. (2018). Baseline sensitivity of *Colletotrichum acutatum* isolates from Brazilian strawberry fields to azoxystrobin, difenoconazole, and thiophanate-methyl. Trop Plant Pathol. 43, 533–542. doi: 10.1007/s40858-018-0232-2

[ref3] Baroncelli R. Sarrocco S. Zapparata A. Tavarini S. Angelini L. G. Vannacci G. (2015). Characterization and epidemiology of *Colletotrichum acutatum* sensu lato (*C. chrysanthemi*) causing *C. arthamus* tinctorius anthracnose. Plant Pathol. 64, 375–384. doi: 10.1111/ppa.12268

[ref4] Baroncelli R. Sreenivasaprasad S. Lane C. R. Thon M. R. Sukno S. A. (2014). First report of *Colletotrichum acutatum* sensu lato (*Colletotrichum godetiae*) causing anthracnose on grapevine (*Vitis vinifer*a) in the United Kingdom. New Disease Reports 29, 26. doi: 10.5197/j.2044-0588.2014.029.026

[ref5] Baroncelli R. Talhinhas P. Pensec F. Sukno S. A. Le Floch G. Thon M. R. (2017). The *Colletotrichum acutatum* species complex as a model system to study evolution and host specialization in plant pathogens. Front. Microbiol. 8, 2001. doi: 10.3389/fmicb.2017.02001, PMID: 29075253 PMC5641571

[ref6] Bhunjun C. S. Phukhamsakda C. Jayawardena R. S. Jeewon R. Promputtha I. Hyde K. D. (2021). Investigating species boundaries in *Colletotrichum*. Fungal Divers. 107, 107–127.

[ref7] Børve J. Djønne R. T. Stensvand A. (2010). *Colletotrichum acutatum* occurs asymptomatically on sweet cherry leaves. Eur J of Plant Pathol. 127, 325–332. doi: 10.1007/s10658-010-9597-x

[ref8] Børve J. Stensvand A. (2006a). *Colletotrichum acutatum* overwinters on sweet cherry buds. Plant Dis. 90, 1452–1456. doi: 10.1094/pd-90-1452, PMID: 30780913

[ref9] Børve J. Stensvand A. (2006b). Timing of fungicide applications against anthracnose in sweet and sour cherry production in Norway. Crop Prot. 25, 781–787. doi: 10.1016/j.cropro.2005.10.012

[ref10] Børve J. Stensvand A. (2013). *Colletotrichum acutatum* can establish on sweet and sour cherry trees throughout the growing season. Eur Hortic Sci. 78, 258–266.

[ref11] Bragança C. Junior A. N. Rogério F. Massola Jr N. (2014). First report of anthracnose caused by *Colletotrichum theobromicola* on Barbados cherry (*Malpighia emarginata*) in Brazil. Plant Dis. 98, 1272. doi: 10.1094/pdis-01-14-0099-PDN, PMID: 30699624

[ref12] Cai L. Giraud T. Zhang N. Begerow D. Cai G. Shivas R. G. (2011). The evolution of species concepts and species recognition criteria in plant pathogenic fungi. Fungal Divers. 50, 121–133. doi: 10.1007/s13225-011-0127-8

[ref100] Cai L. Hyde K. Taylor P. Weir B. Waller J. Abang M.. (2009). A polyphasic approach for studying colletotrichum. Fungal Divers. 39, 183–204.

[ref200] Carbone I. Kohn L. M. (1999). A method for designing primer sets for speciation studies in filamentous ascomycetes. Mycologia 91, 553–556. doi: 10.2307/3761358

[ref13] Chen S. Luo C. Hu M. Schnabel G. (2016). Sensitivity of Colletotrichum species, including *C. fioriniae* and *C. nymphaeae*, from peach to demethylation inhibitor fungicides. Plant Dis. 100, 2434–2441. doi: 10.1094/pdis-04-16-0574-re, PMID: 30686167

[ref14] Chen D. Shi H. Wu H. Xu Z. Zhang C. (2013a). Resistance of *Colletotrichum gloeosporioides* causing grape ripe rot to thiophanate-methyl and tebuconazole in Zhejiang. J. Fruit Sci. 30, 665–668.

[ref15] Chen T. Wang X. R. Luo H. Wang C. T. Luo M. M. (2012). Chloroplast DNA trnQ-rps16 variation and genetic structure of nine wild Chinese cherry (*Cerasus pseudocerasus* Lindl.) populations. Hereditas (Beijing) 34, 1475–1483. doi: 10.3724/sp.j.1005.2012.01475, PMID: 23208145

[ref16] Chen T. Wang X. R. Tang H. R. Chen Q. Huang X. J. Chen J. (2013b). Genetic diversity and population structure of Chinese cherry revealed by chloroplast DNA trnQ-rps16 intergenic spacers variation. Genet. Resour. Crop Evol. 60, 1859–1871. doi: 10.1007/s10722-013-9960-9

[ref17] Chethana K. Jayawardene R. Zhang W. Zhou Y. Liu M. Hyde K.. (2019). Molecular characterization and pathogenicity of fungal taxa associated with cherry leaf spot disease. Mycosphere 10, 490–530. doi: 10.5943/mycosphere/10/1/8

[ref18] Crous P. W. Groenewald J. Z. Risede J. Hyweljones N. (2004). *Calonectria* species and their *cylindrocladium anamorphs*: species with sphaeropedunculate vesicles. Stud. Mycol. 157, 127–135. doi: 10.1023/b:myco.0000012225.79969.29 PMC2104717 18490981

[ref19] Da Lio D. Cobo-Díaz J. F. Masson C. Chalopin M. Kebe D. Giraud M.. (2018). Combined metabarcoding and multi-locus approach for genetic characterization of *Colletotrichum* species associated with common walnut (*Juglans regia*) anthracnose in France. Sci. Rep. 8, 1–17. doi: 10.1038/s41598-018-29027-z, PMID: 30018385 PMC6050315

[ref20] Damm U. Cannon P. Woudenberg J. Crous P. (2012). The *Colletotrichum acutatum* species complex. Stud. Mycol. 73, 37–113. doi: 10.3114/sim0010, PMID: 23136458 PMC3458416

[ref21] Darriba D. Taboada G. L. Doallo R. Posada D. (2012). jModelTest 2: more models, new heuristics and parallel computing. Nat. Methods 9, 772. doi: 10.1038/nmeth.2109, PMID: 22847109 PMC4594756

[ref22] Dean R. Van Kan J. A. Pretorius Z. A. Hammond-Kosack K. E. Di Pietro A. Spanu P. D.. (2012). The top 10 fungal pathogens in molecular plant pathology. Mol. Plant Pathol. 13, 414–430. doi: 10.1111/j.1364-3703.2012.00822.x, PMID: 22471698 PMC6638784

[ref23] Etebarian H. R. Sholberg P. L. Eastwell K. C. Sayler R. J. (2005). Biological control of apple blue mold with *Pseudomonas fluorescens*. Can. J. Microbiol. 51, 591–598. doi: 10.1139/w05-039, PMID: 16175208

[ref24] Forcelini B. B. Rebello C. S. Wang N. Y. Peres N. (2017). Fitness, competitive ability and mutation stability of isolates of *Colletotrichum acutatum* from strawberry resistant to qoi fungicides. Phytopathology 108, 462–468. doi: 10.1094/phyto-09-17-0296-R 29135359

[ref25] Forcelini B. B. Seijo T. E. Amiri A. Peres N. A. (2016). Resistance in strawberry isolates of *Colletotrichum acutatum* from Florida to quinone-outside inhibitor fungicides. Plant Dis. 100, 2050–2056. doi: 10.1094/pdis-01-16-0118-re, PMID: 30683005

[ref26] Hall T. A. (1999). Bioedit: a user-friendly biological sequence alignment editor and analysis program for windows 95/98/nt. Nuclc Acids Symposium Series 734, 95–98. doi: 10.1021/bk-1999-0734.ch008

[ref27] Hu M. J. Grabke A. Dowling M. E. Holstein H. J. Schnabel G. (2015). Resistance in *Colletotrichum siamense* from peach and blueberry to thiophanate-methyl and azoxystrobin. Plant Dis. 99, 806–814. doi: 10.1094/pdsi-10-14-1077-RE, PMID: 30699530

[ref28] Inada M. Ishii H. Chung W. H. Yamada T. Yamaguchi J. I. Furuta A. (2008). Occurrence of strobilurin-resistant strains of *Colletotrichum gloeosporioides* (*Glomerella cingulata*), the causal fungus of strawberry anthracnose. Jpn. J. Phytopathol. 74, 114–117. doi: 10.3186/jjphytopath.74.114

[ref29] Jayawardena R. S. Bhunjun C. S. Hyde K. D. Gentekaki E. Itthayakorn P. (2021). *Colletotrichum*: lifestyles, biology, morpho-species, species complexes and accepted species. Mycosphere 12, 519–669. doi: 10.5943/mycosphere/12/1/7

[ref30] Jayawardena R. Hyde K. Damm U. Cai L. Liu M. Li X.. (2016). Notes on currently accepted species of *Colletotrichum*. Mycosphere 7, 1192–1260. doi: 10.5943/mycosphere/si/2c/9

[ref31] Ji M. Wu X. Yao K. Chen H. Yang J. Wang L.. (2014). Identification of strawberry anthracnose pathogens and screening of germicides. Agric. Sci. Technol. 15, 94.

[ref32] Katoh K. Standley D. M. (2013). MAFFT multiple sequence alignment software version 7: improvements in performance and usability. Mol. Biol. Evol. 30, 772–780. doi: 10.1093/molbev/mst010, PMID: 23329690 PMC3603318

[ref33] Kim C. H. Hassan O. Chang T. (2020). Diversity, pathogenicity, and fungicide sensitivity of *Colletotrichum* species associated with apple anthracnose in South Korea. Plant Dis. 104, 2866–2874. doi: 10.1094/PDIS-01-20-0050-RE 32924872

[ref34] Kim D. O. Heo H. J. Kim Y. J. Yang H. S. Lee C. Y. (2005). Sweet and sour cherry phenolics and their protective effects on neuronal cells. J. Agr. Food Chem. 53, 9921–9927. doi: 10.1021/jf0518599, PMID: 16366675

[ref35] Kim S. Min J. Back D. Kim H. Lee S. Kim K. (2016). Assessment of QoI resistance in *Colletotrichum* spp. isolated from boxthorn and apple in Korea. Phytopathology 48, 6–71. doi: 10.1016/j.cimid.2016.07.001

[ref36] Kongtragoul P. Imamoto K. Ishii H. (2020). Resistance to quinone-outside inhibitor (QoI) fungicides in *Colletotrichum* species isolated from anthracnose disease occurring in Thailand. Curr. Appl. Sci. Technol. 48, 6–13. doi: 10.1016/j.cimid.2016.07.001

[ref37] Li H. X. Liu Z. Y. Wang J. X. Zhou M. G. (2005). Baseline sensitivity of *Colletotrichum gloeosporioides* and *C. capsici* from capsium to azoxystrobin. Acta Phytopathol. Sin. 35, 73–77.

[ref38] Liu Y. An F. Zhang Y. Fu C. Su Y. (2021). First report of anthracnose on Jerusalem Cherry caused by *Colletotrichum liaoningense* in Shandong, China. Plant Dis. 105:2248. doi: 10.1094/pdis-01-21-0124-PDN

[ref39] López-Moral A. Raya-Ortega M. C. Agustí-Brisach C. Roca L. F. Lovera M. Luque F.. (2017). Morphological, pathogenic, and molecular characterization of *Colletotrichum acutatum* isolates causing almond anthracnose in Spain. Plant Dis. 101, 2034–2045. doi: 10.1094/pdis-03-17-0318-re, PMID: 30677386

[ref40] Miller M. A. Pfeiffer W. Schwartz T. (2010). “Creating the CIPRES science gateway for inference of large phylogenetic trees.” in *2010 Gateway Computing Environments Workshop (GCE)*, New Orleans, LA, 1–8.

[ref41] Mo F. Hu X. Ding Y. Li R. Li M. (2021). Naturally produced magnolol can significantly damage the plasma membrane of Rhizoctonia solani. Pestic. Biochem. Physiol. 178:104942. doi: 10.1016/j.pestbp.2021.104942, PMID: 34446208

[ref42] Mosca S. Li Destri Nicosia M. G. Cacciola S. O. Schena L. (2014). Molecular analysis of *Colletotrichum* species in the carposphere and phyllosphere of olive. PloS One 9:e114031. doi: 10.1371/journal.pone.0114031, PMID: 25501572 PMC4263604

[ref43] Munda A. (2014). First report of *Colletotrichum fioriniae* and *C. godetiae* causing apple bitter rot in Slovenia. Plant Dis. 98, 1282. doi: 10.1094/pdis-04-14-0419-PDN, PMID: 30699646

[ref400] O’Donnell K. Cigelnik E. (1997). Two divergent intragenomic rdna its2 types within a monophyletic lineage of the fungus fusarium are nonorthologous. Mol. Phylogenet. Evol. 7, 103–116. doi: 10.1006/mpev.1996.0376 9007025

[ref500] Rambaut A. (2016). FigTree version v1.4.3 [online]. Available at: https://github.com/rambaut/figtree/releases/tag/v1.4.3 (Accessed October 04, 2016).

[ref44] Ronquist F. Teslenko M. Van Der Mark P. Ayres D. L. Darling A. Höhna S.. (2012). MrBayes 3.2: efficient Bayesian phylogenetic inference and model choice across a large model space. Syst. Biol. 61, 539–542. doi: 10.1093/sysbio/sys029, PMID: 22357727 PMC3329765

[ref45] Sharma G. Kumar N. Weir B. S. Hyde K. D. Shenoy B. D. (2013). The ApMat marker can resolve *Colletotrichum* species: a case study with *Mangifera indica*. Fungal Divers. 61, 117–138. doi: 10.1007/s13225-013-0247-4

[ref46] Shi N. Ruan H. Gan L. Dai Y. Yang X. Du Y.. (2020). Evaluating the sensitivities and efficacies of fungicides with different modes of action against *Phomopsis asparagi*. Plant Dis. 104, 448–454. doi: 10.1094/pdis-05-19-1040-RE, PMID: 31801035

[ref47] Shi N. N. Ruan H. C. Jie Y. L. Chen F. R. Du Y. X. (2021). Characterization, fungicide sensitivity and efficacy of *Colletotrichum* spp. from chili in Fujian. China. Crop Prot. 143:105572. doi: 10.1016/j.cropro.2021.105572

[ref600] Stamatakis A. (2014). RAxML version 8: a tool for phylogenetic analysis and post - analysis of large phylogenies. Bioinformatics 30, 1312–1313. doi: 10.1093/bioinformatics/btu033 24451623 PMC3998144

[ref48] Stensvand A. Børve J. Talgø V. (2017). Overwintering diseased plant parts and newly infected flowers and fruit as sources of inoculum for *Colletotrichum acutatum* in sour cherry. Plant Dis. 101, 1207–1213. doi: 10.1094/pdis-11-16-1599-re, PMID: 30682962

[ref700] Talhinhas P. Gonçalves E. Sreenivasaprasad S. Oliveira H. (2015). Virulence diversity of anthracnose pathogens (*Colletotrichum acutatum* and *C. gloeosporioides* species complexes) on eight olive cultivars commonly grown in Portugal. Eur. J. Plant. Pathol 142, 73–83. doi: 10.1007/s10658-014-0590-7

[ref49] Tang Z. Lou J. He L. Wang Q. Chen L. Zhong X.. (2021). First report of *Colletotrichum fructicola* causing anthracnose on cherry (*Prunus avium*) in China. Plant Dis. 106, 317. doi: 10.1094/pdis-03-21-0544-pdn

[ref800] Templeton M. D. Rikkerink E. H. A. Solon S. L. Crowhurst R. (1992). Cloning and molecular characterization of the glyceraldehyde-3-phosphate dehydrogenase-encoding gene and cDNA from the plant pathogenic fungus glomerella cingulata. Gene. 122, 225–230. doi: 10.1016/0378-1119(92)90055-t 1452034

[ref50] Wang Q. H. Fan K. Li D. W. Han C. M. Qu Y. Y. Qi Y. K.. (2020). Identification, virulence and fungicide sensitivity of *Colletotrichum gloeosporioides* s. s. Responsible for walnut anthracnose disease in China. Plant Dis. 104, 1358–1368. doi: 10.1094/pdis-12-19-2569-re, PMID: 32196416

[ref51] Wang Q. H. Fan K. Li D. W. Niu S. G. Hou L. Q. Wu X. Q. (2017). Walnut anthracnose caused by *Colletotrichum siamense* in China. Australas. Plant Path. 46, 585–595. doi: 10.1007/s13313-017-0525-9

[ref900] Wang X. H. Wang R. Fa L. Zhang Y. A. Wang H. X. Liu X.. (2019). Pathogen dentification of anthracnose on juglans regia in shaanxi province. J. Northeast For. Univ. 47, 113–119. doi: 10.13759/j.cnki.dlxb.2019.11.022

[ref1000] White T. J. Bruns T. Lee S. Taylor J. W. (1990). “Amplification and direct sequencing of fungal ribosomal RNA genes for phylogenetics”, in PCR protocols: a guide to methods and applications. 315–322.

[ref52] Wijayawardene N. Hyde K. Al-Ani L. Tedersoo L. Haelewaters D. Rajeshkumar K. (2020). Outline of fungi and fungus-like taxa. Mycosphere 11, 1060–1456. doi: 10.5943/mycosphere/11/1/8

[ref53] Wijayawardene N. N. Hyde K. D. Lumbsch H. T. Liu J. K. Maharachchikumbura S. S. Ekanayaka A. H.. (2018). Outline of ascomycota: 2017. Fungal Divers. 88, 167–263. doi: 10.1007/s13225-018-0394-8

[ref54] Yokosawa S. Eguchi N. Kondo K. I. Sato T. (2017). Phylogenetic relationship and fungicide sensitivity of members of the *Colletotrichum gloeosporioides* species complex from apple. J. Gen. Plant Pathol. 83, 291–298. doi: 10.1007/s10327-017-0732-9

[ref55] Yuan X. Peng K. Li C. Zhao Z. Zeng X. Tian F.. (2021). Complete genomic characterization and identification of *Saccharomycopsis phalluae* sp. nov., a novel pathogen causes yellow rot disease on *phallus rubrovolvatus*. J Fungi. 7, 707. doi: 10.3390/jof7090707, PMID: 34575745 PMC8468998

[ref1100] Zhang X. Y. Li X. Gao Z. Y. (2014). Carbendazim resistance of *Colletotrichum gloeosporioides* on tropical and subtropical fruits. Chinese J. Tropical Agr. 34, 71–74.

[ref56] Zhang L. Song L. Xu X. Zou X. Duan K. Gao Q. (2020). Characterization and fungicide sensitivity of *Colletotrichum* species causing strawberry anthracnose in eastern China. Plant Dis. 104, 1960–1968. doi: 10.1094/pdis-10-19-2241-re, PMID: 32401619

[ref57] Zhang Q. Yan G. Dai H. Zhang X. Li C. Zhang Z. (2008). Characterization of tomentosa cherry (*Prunus tomentosa* Thunb.) genotypes using SSR markers and morphological traits. Sci Hortic-Amsterdam. 118, 39–47. doi: 10.1016/j.scienta.2008.05.022

